# Connection Confinement of Bolted Fibre-Reinforced Polymer Bamboo Composite

**DOI:** 10.3390/polym14102051

**Published:** 2022-05-17

**Authors:** Joel Kennaway, Ali Rajabipour, Dongsheng Huang, Milad Bazli, Siyuan Tang, Junkai Wang, Hayden Zanker, Fangming Su

**Affiliations:** 1 College of Engineering, IT & Environment, Charles Darwin University, Darwin 0801, Australia; joel.kennaway2@students.cdu.edu.au (J.K.); hayden.zanker@students.cdu.edu.au (H.Z.); fsu@njfu.edu.cn (F.S.); 2 National Engineering Research Center of Biomaterials, Nanjing Forestry University, Nanjing 210037, China; dshuang@njfu.edu.cn (D.H.); stang@njfu.edu.cn (S.T.); jwang@njfu.edu.cn (J.W.); 3 School of Mechanical and Mining Engineering, The University of Queensland, Brisbane 4000, Australia

**Keywords:** bamboo, bolted connection, composite, confinement, damage, failure, shear-out, strength

## Abstract

Parallel strand bamboo is a composite material that demonstrates high strength and low variability compared to other timber materials. However, its use in bolted connections is limited by a tendency to fail in shear-out mode. One promising technique to prevent failure is the method of confinement, whereby the composite connection is confined laterally, inducing a compressive force perpendicular to the composite fibres, which increases the shear strength in the loading process. This paper investigates the confinement method and its effect on parallel strand bamboo connections’ strength and failure mechanisms through experimental tests and ANSYS simulation methods. It was discovered that bolted connection confinement reduces the propensity of shear-out failure by counteracting shear stresses. A comparison of graphical results revealed that confinement increased the ultimate tensile capacity of parallel strand bamboo bolted connections by up to 26%. Confinement also improved the consistency of the connection’s mechanical properties throughout the loading process. These findings assist in refining and optimising practical applications of parallel strand bamboo connections by using the method of connection confinement.

## 1. Introduction

### 1.1. Parallel Strand Bamboo

Bamboo is an abundant, versatile material with a strength to weight ratio greater than structural steel and material properties that tend to be superior to other natural timbers [1,2,3,4,5,6,7]. These characteristics present promising opportunities to utilise bamboo as a structural material. One of the contributors to the high stiffness and strength of bamboo materials is its small microfibrillar angle between 2° and 10°, which is known to have a critical impact on the mechanical properties of bamboo, predominantly its stiffness [8,9,10,11,12,13]. Bamboo’s small microfibrillar angle and high resulting tensile capacity make it a suitable choice of reinforcing material in various composites [11,12]. The recent development of bamboo composites promises to expand the use of bamboo as a structural material [14,15,16,17].

**Figure 1:**
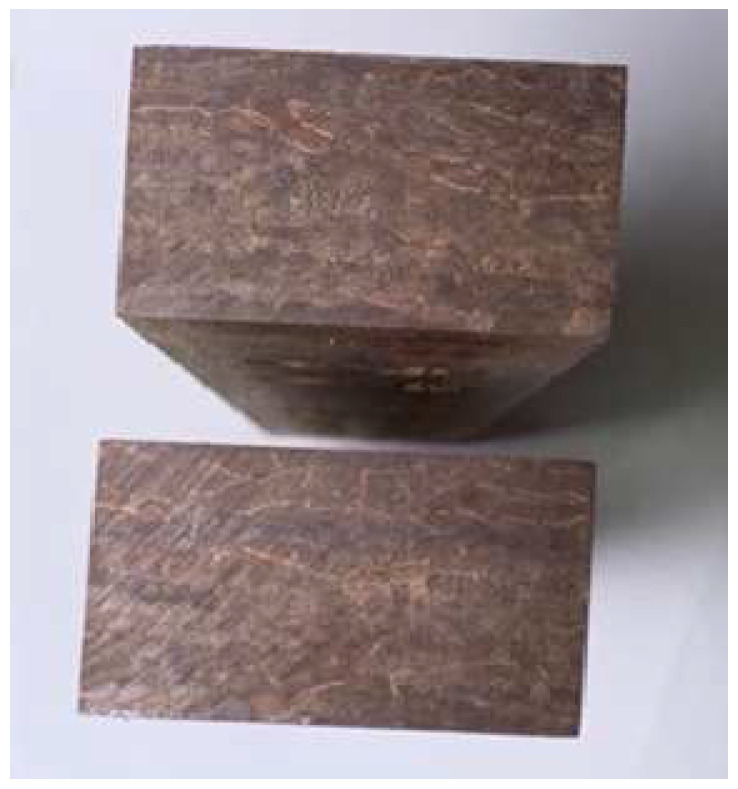
Parallel Strand Bamboo Cross Section.

One recently developed bamboo-composite is Parallel Strand Bamboo (PSB), which demonstrates greater strength properties than most timber materials [18]. Samples of the material are shown in Figure 1.

PSB is a unidirectional fibre composite where the long fibres are made from natural bamboo strands. The remaining material volume consists of a matrix bonded to the fibres with an adhesive made from phenolic resin [18,19]. Fibres in PSB are extracted from Phyllostachys, a common bamboo species in South China. Bamboo culms are cut in strips of 2 m × 15 mm × 3 mm and dried at 80 °C prior to being flattened and impregnated with phenolic resin and finally pressed [18]. The final product has an average density of 1100 kg/m^3^ [18]. Due to its unidirectional nature, when PSB is loaded along the axis of its fibres, all fibres are recruited in the loading process, resulting in a higher tensile capacity compared to multidirectional composites [20,21,22]. However, when PSB is loaded in tension perpendicular to the fibres’ orientation, it demonstrates significantly less capacity than a multidirectional composite since the fibres are not directly recruited to carry the load. As such, PSB’s unique strength in tension parallel to the fibres is much higher than perpendicular to the fibres. This is reflected in the elastic moduli of PSB; one study revealed the modulus parallel to the fibres was 10.30 GPa, while the elastic modulus perpendicular to the fibres was much lower at 2.22 GPa [18]. Moreover, strength tests have confirmed that PSB’s tensile strength parallel to the fibres is significantly larger than perpendicular to the fibres by over three times [18]. The tensile strength perpendicular to the fibres depends on the matrix’s relatively low tensile strength [18]. In practice, the high strength of the fibres in comparison to the matrix tends to cause the matrix to fail before the fibres rupture. When compressed parallel to the fibres, the fibres tend to buckle laterally at relatively low loads, while compression perpendicular to the fibres results in the fibres restricting transverse deformations and increasing strength [18]. This finding is particularly relevant to the intent of this project since it suggests that lateral confinement may reduce this type of lateral buckling of fibres by restricting the transverse movement of the fibres and reinforcing the material so that strength is not dependent upon the tensile strength of the matrix alone. PSB shear strength parallel to the fibres is also lower than the strength perpendicular to the fibres by a factor of approximately three.

A common practice to mechanically connect composite materials is bolted connections. Although they are the most efficient method of mechanical joining [23], bolted connections introduce significant stress concentrations surrounding the bolt holes in composite materials [20,23,24,25,26,27,28,29]. The way these stress concentrations are redistributed depends on whether the material is ‘notch sensitive’ or ‘insensitive’ [30]. Composites are generally notch-sensitive, with a limited ability to redistribute loads around notches [30,31,32,33]. Given the limited capacity of composites to redistribute stresses around bolt holes, stress concentrations must be considered in the design process of connections to avoid catastrophic connection failure. The strength of single bolted connections to resist shear-out failure mode depends on ply orientations within the material, with single ply-oriented, unidirectional materials generally demonstrating lower strength than multidirectional laminates [23]. Since PSB in bolted connections is vulnerable to shear-out failure and other failure mechanisms, a method to avoid these significant vulnerabilities must be established to expand PSB’s potential use as a structural material.

### 1.2. Failure Modes

**Figure 2:**
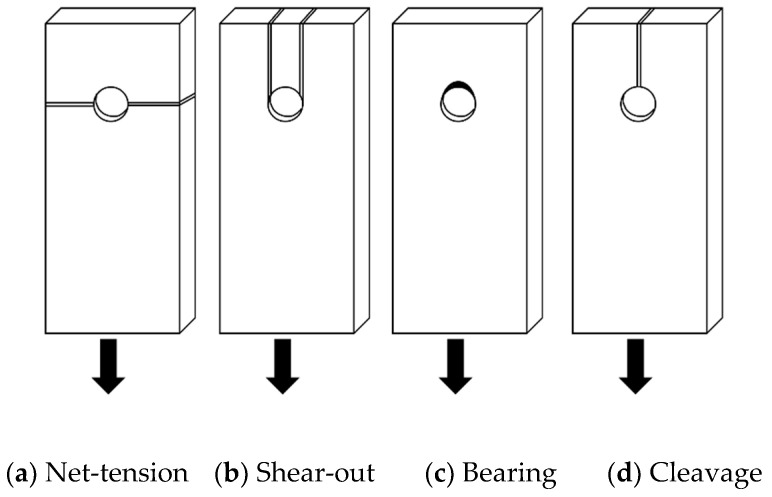
Failure Modes of Bolted Composite Plates.

Figure 2 illustrates the four typical modes of failure for composite plate bolted connections: (a) net-tension, (b) shear-out, (c) bearing and (d) cleavage failure.

Net-tension failure is typically the result of excessive tensile stress along the failure cross-section [34,35]. In contrast, shear-out failure occurs when high shear stresses cause the section ahead of the bolt to punch through the composite connection [34,36]. Bearing failure of bolted connections propagates gradually and occurs when excessive compressive stress is imparted at the contact surface between bolt and bolt hole [20,34,37,38,39,40,41]. Cleavage failure is a combination of failure modes whereby the material typically splits due to tensile stresses perpendicular to the fibre orientation [40]. Given that the tensile strength of PSB parallel to the fibres is much stronger than perpendicular to the fibres, it is expected that PSB connections will predominantly fail in either cleavage mode due to tensile stresses developed perpendicular to the fibres or shear-out mode due to shear stresses developed along either side of individual bolts or bolt groups. This is supported by studies that have found that connection failures using timber with properties similar to PSB tended to occur through the process of cleavage failure, where a single crack began at maximum perpendicular to grain tensile stresses at the edge of the bolt hole and progressed along the grain direction [14,31]. While the causes of failures in composite connections are evident, methods to prevent these modes of failure in PSB bolted connections, particularly the expected critical mode of shear-out failure, require further investigation.

### 1.3. Strengthening Methods

A literature review has highlighted several composite connection-strengthening methods that have been investigated. These methods include enhancing geometry configuration, improving composite material properties, and adding external features to connections.

Investigations regarding enhancing geometry configuration have experimented with various combinations of composite thicknesses and pin diameters in composite joints. It was found that joint stiffness and strength were reduced in thick composites with small pin diameters and thin composites with larger pin diameters [42]. The configurations with similarly proportioned connection thickness and pin diameter dimensions demonstrated better stiffness and strength properties, guiding the design of bolted composite connection dimensions. Additionally, it has been noted that an increase in bolt hole clearance reduces bearing contact area, increasing stress and negatively impacting joint strength [20]. Moreover, when analysing connections with a three-dimensional finite element analysis (FEA) model, it was found that bolt clearance significantly affected stress distribution around bolt holes, and the load of failure initiation and fatigue life were impacted by the clearance range [43,44,45]. In a separate study, the behaviour of bolted connections using laminated bamboo and steel plates was investigated and found that bolt spacing and configuration played a vital role in determining the strength of multiple bolt connections [46].

Several research projects have focused on enhancing composite material properties to improve connection strength, including research regarding reducing stress concentrations around bolt holes by creating composites with variable stiffness and curved fibre orientations [47]. It has been found that ultimate connection capacity is significantly enhanced when fibres surrounding the bolt hole are oriented at optimal angles forming concentric rings near the hole and gradually merging with fibres further from the hole [48]. Supporting this, the curved orientation of curvilinear fibre plies surrounding boltholes in carbon fibre reinforced polymer (CFRP) composites has also been shown to increase composite fracture strength [49,50]. Other papers have shown that curvilinear fibre orientations in composites tend to shift the stress concentration from the area surrounding the hole to the edges of the plate, thus increasing the composite’s buckling capacity and enhancing tensile capacity as a result [51]. 

Furthermore, the testing results of 3D printed continuous curved fibre composites with variable stiffness and fibre volume fraction have been compared to the properties of linear fibre orientations in traditional composites [52]. The results highlighted a significant enhancement of stiffness and strength per unit weight of up to 9.4 and 1.6 times, respectively, indicating that deviation from traditional linear composite manufacturing may be beneficial to the strengthening of future composite materials. When investigating the optimisation of bolt hole arrangement in composites using manufacturing techniques to achieve variable and constant stiffness, it was found that stress concentrations surrounding the hole decreased in the variable stiffness designs. The research indicates that composite strength can be enhanced by achieving variable stiffness through advanced fibre orientation manufacturing processes [53,54]. Separate studies have shown that connection capacity can be improved by adjusting the parameters of connection materials in fibre reinforced beam-to-column connections in laminated timber frames. 

Dowel bearing strength tended to increase as the density of the connected timber material increased [55]; however, the process of densifying material led to micro-damage and a higher tendency for brittle failure. To prevent this, it was identified that connections could be reinforced with materials such as attaching glass fibre fabric or densified veneer wood disks. However, this presents further issues regarding creep, shrinkage, and differential thermal expansion, which would need to be considered [55]. Microscopic material enhancement has also been investigated, incorporating carbon nanotubes in prestressed glass fibre reinforced polymer shown to increase shear strength, ductility and tensile strength by enhancing the bond between matrix and fibres [56]. Similarly, incorporating ceramic nanoparticles into a composite material’s matrix has been observed to result in a shear strength increase due to additional particles at interlaminar regions [57].

The effect of adding external features to composite connections has also been investigated. For example, the incorporation of metal inserts in fibre reinforced polymer composite bolted joints have been shown to reduce the significant stress concentrations experienced in the region surrounding the bolt hole, thereby delaying the onset of damage initiation. The metal inserts had the effect of bolt tension relaxation in the composite, resulting in increased load capacity in the connection before material damage began to occur. This suggests traditional bolt holes can be optimised with the incorporation of metal inserts [58]. Additionally, pre-tensioning of connection bolts has been shown to provide lateral constraint to the region beneath the bolt, increasing joint strength and load-carrying capacity [40]. However, excess pressure has been found to potentially induce delamination in the area and reduce strength [59].

The general findings of the literature review regarding connection strengthening methods found that attempts to increase the strength of connections predominantly focus on enhancing the geometry of connection configurations, and optimising connection material properties, usually resulting in the formulation of new and improved composite materials. These methods focus on internal material adjustments. However, fewer studies exist regarding the use of external factors and adjustments to improve the strength of composite connections without altering the material itself. This project will investigate an external method of enhancing the strength of bolted composite connections, known as the method of connection confinement.

### 1.4. Connection Confinement

**Figure 3:**
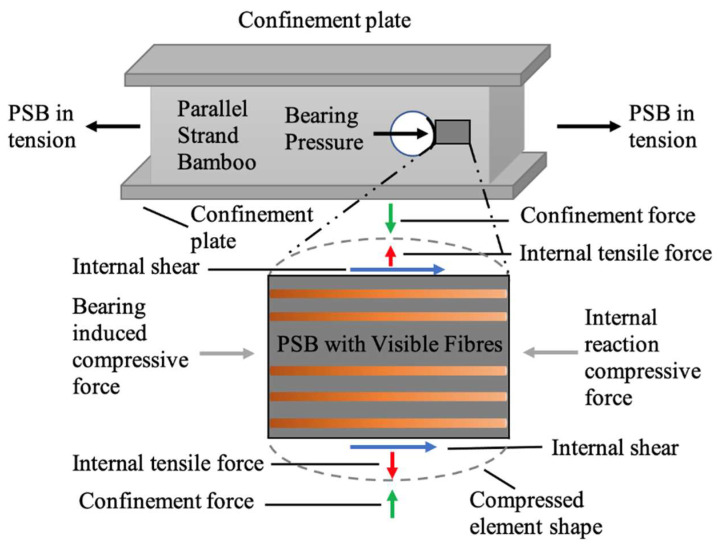
Schematic of internal stresses in PSB connection with confinement.

A promising external method of prolonging connection failure and enhancing ultimate capacity is connection confinement. This involves laterally confining a composite connection with wrapped materials such as carbon fibre reinforced polymer (CFRP) or glass fibre reinforced polymer (GFRP). This method hypothesises that confinement restricts lateral displacement and induces a compressive force perpendicular to the composite fibres, opposing transverse tensile stresses and reducing perpendicular shear stresses generated in the loading process. To investigate this method, this experiment used confinement plates to impose compressive stress transverse to the axis of fibre orientation. This is demonstrated schematically through the setup shown in Figure 3, where a PSB connection is loaded in tension along the axis of its fibres, and steel plates are used to represent the confining material parallel to the fibres. A segment ahead of the bolt hole is shown to represent the internal stresses and illustrate the confinement effect.

As shown in Figure 3, the tensile load causes the bolt to press against the PSB and induces a bearing pressure on the edge of the bolt hole, and shear stresses along the side of the hole. These internal tensile and shear forces can be critical for a unidirectional composite material such as PSB, which has a relatively weak matrix tensile and shear strength in this direction [60]. If these tensile and shear forces are not accounted for, they can cause either cleavage or shear-out failure to occur. However, in a confined connection, the internal tensile forces developed are expected to be opposed by the compressive forces (shown in green in Figure 3) induced by the confinement pressure. Furthermore, the shear stresses are reduced by imposing additional normal pressure perpendicular to the fibres, thus enhancing the shear capacity of the connection.

Given that PSB has shown a tendency to fail at low tensile and shear stresses, it is hypothesised that this method of connection confinement will reduce the propensity of cleavage and shear-out failure and increase the loading capacity of PSB bolted connections [61]. Recent research has investigated the effect of connection confinement achieved by wrapping single bolt timber connections with CFRP as a method of timber strengthening [62]. Overall, the study demonstrated that CFRP confinement could increase the strength of softwood connections by as much as 58% and hardwood connections by up to 50%, giving confidence that a confining material in PSB connections may increase connection strength. Similarly, a separate study found that external FRP reinforcement increased capacity in some connections by up to 60% [63]. Additionally, a distinct method of reinforcing confinement involving GFRP bolted connections were investigated to increase connection shear capacity [64]. The confinement setup involved layered composite reinforcing with glass fibre sheets (GFS) adhered to the material on the bolt hole surface. The research found that GFS reinforcing could significantly increase the ultimate capacity and stiffness of GFRP connections, with ultimate loading increased by up to 2.6 times and joint stiffness increased by almost 90%. These three studies that employ a type of confinement strengthening mechanism support the hypothesis that confinement may also increase strength in PSB connections.

This study investigates the effect of confinement on improving the strength and failure mechanism of PSB bolted connections. In this paper, an approach to practically investigate the effect of confinement is outlined and conducted. A simulation model of the unconfined experimental setup is then created using ANSYS and is verified by the presented experimental data. The model is then used to clarify experimental observations while the results are discussed to identify the effect of confinement on connection strength. The suitability of the developed numerical model for future investigation is illustrated, and finally, the concluding implications from the results are identified.

## 2. Experimental Approach

**Figure 4:**
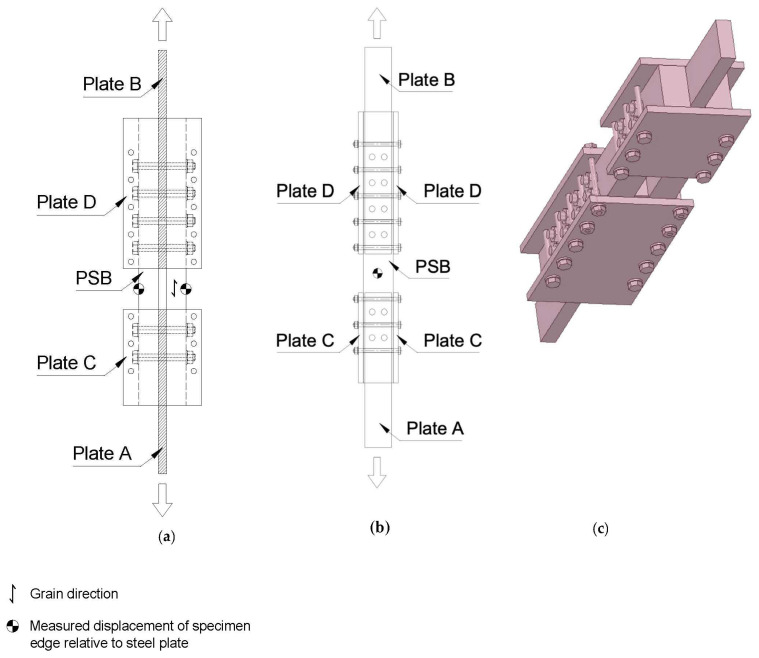
(**a**) Side view; (**b**) top view; (**c**) confinement test-setup 3D view.

**Figure 5:**
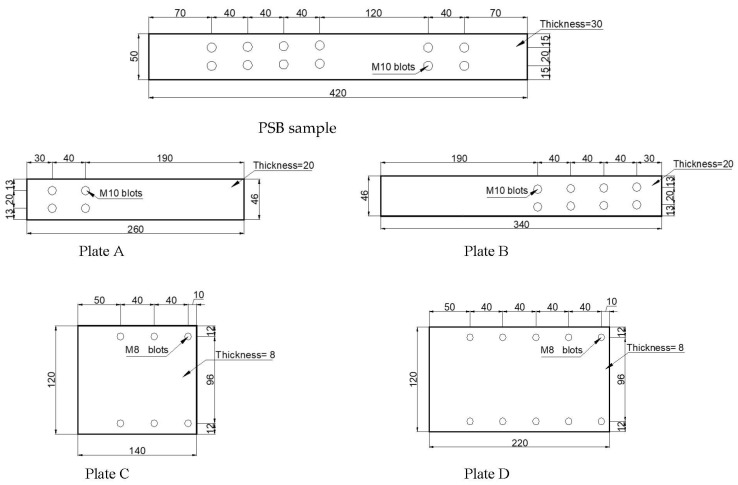
Sizes and details of the plates labelled in Figure 4.

The experimental approach involved conducting tests of tensile capacity and failure mode of bolted connections at different levels of confinement force. The detailed arrangement of the test connection with a 3D view is shown in Figure 4. Details of the connection plates are shown in Figure 5. 

**Figure 6:**
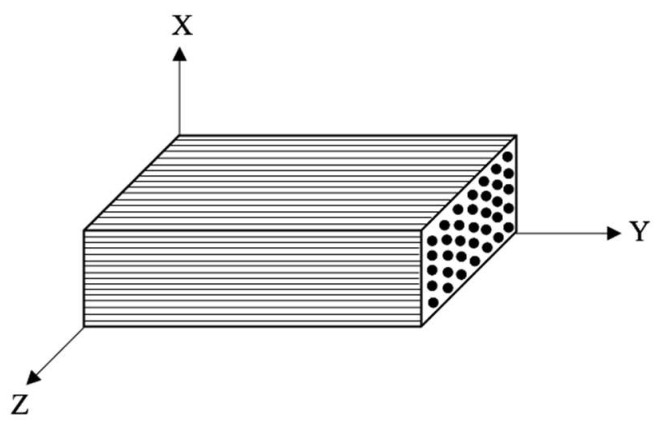
PSB Axis Legend.

The strength properties of PSB were investigated using ASTM Tests D143-09 and D7078 [18]. The properties found through the tests are summarised in Table 1, where composite fibres are oriented on the *y*-axis according to the axis legend in Figure 6.

M10 bolts of grade 10.9 were used to connect the PSB to the steel loading plates A and B, which had an ultimate tensile strength of 1100 MPa. Four different levels of confinement pressure were induced by a certain amount of torque in the M8 bolts holding Plates C and D in place. The confinement pressures were 0 MPa (i.e., no confinement pressure), 1.8 MPa, 4.5 MPa, and 8.9 MPa. A 2000 kN universal testing machine was then used to progressively apply a tensile load to the steel loading plate, Plate A, at a speed of 1 mm per minute until the point of connection failure. The end of Plate B was clamped to represent a fixed support. Laser sensors and strain gauges in conjunction with a data logger were used to record the reaction force and corresponding displacement automatically. The resulting deflection at different load increments was graphically represented in load–relative displacement plots.

## 3. Experimental Results

A summary of the experimental test results is shown in Table 2.

### 3.1. Unconfined PSB Connection (0 MPa)

**Figure 7:**
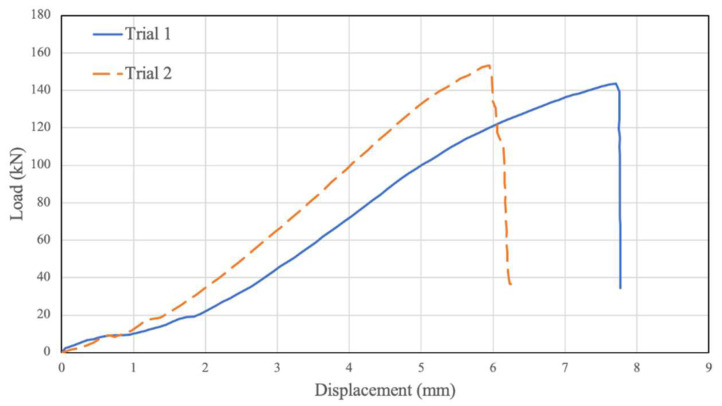
Unconfined 0 MPa Displacement vs. Load.

**Figure 8:**
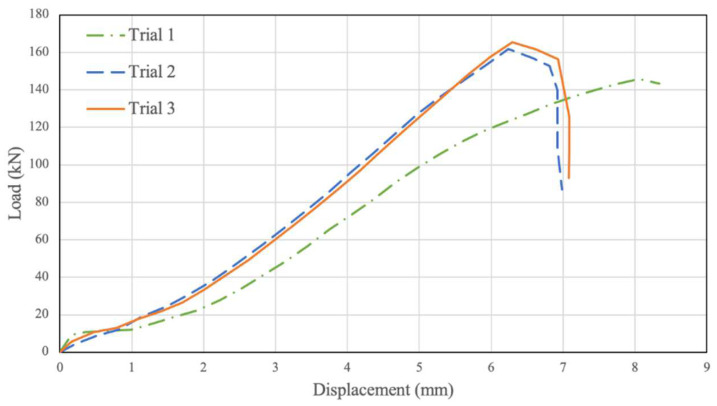
Confined 1.8 Mpa. Displacement vs. Load.

Two testing trials were conducted when investigating the behaviour of the unconfined PSB connections, then were plotted in Figure 7. As seen in the figure, the load–relative displacement graph initially increases in an approximately linear fashion until a displacement of 1.5 mm to 2 mm and a load of 20 kN. At this point, the graph’s slope rises significantly, and the results continue linearly along this slope until a displacement of 5 mm to 5.5 mm and a load of 130 kN to 150 kN. Beyond this point, the slope of the graph decreases until the point of failure, which is a 7.20 mm displacement and 150.61 kN ultimate load on average. Variations in the results for different trials in Figure 7 and Figure 8 could be associated with the variation of mechanical properties in natural fibres. Further, our observations of similar samples show that, sometimes, resin does not penetrate thoroughly between bamboo strands and, therefore, can make a random local imperfection in the material.

### 3.2. Confined PSB Connection (1.8 Mpa)

Three testing trials were conducted to investigate the PSB connections’ behaviour with 1.8 Mpa of confinement pressure, and the resulting data were plotted in Figure 8. Similar to the unconfined results, the load–relative displacement graph initially increases in an approximately linear fashion until a displacement of 1.5 mm to 2 mm and a load of 20 kN to 30 kN. At this point, the graph’s slope increases, and the results continue linearly along this slope until a displacement of 5.5 mm and a load of 150 kN. Beyond this point, the slope of the graph decreases slightly until the average point of failure at 7.17 mm displacement and 162 kN ultimate load.

### 3.3. Confined PSB Connection (4.5 Mpa)

**Figure 9:**
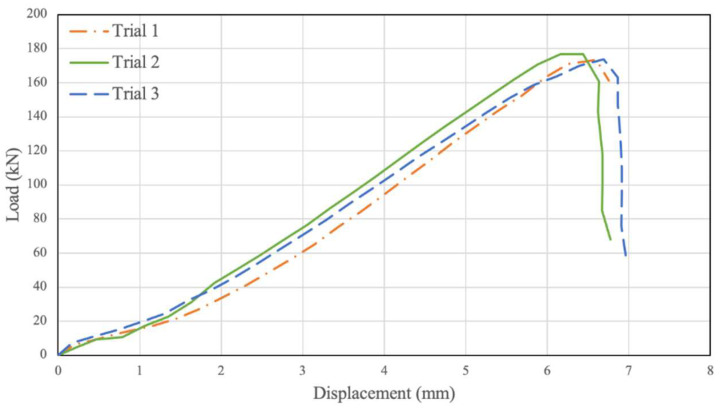
Confined 4.5 Mpa. Displacement vs. Load.

Another three testing trials were conducted when investigating the behaviour of the PSB connections confined with a pressure of 4.5 Mpa, and the resulting data were plotted in Figure 9. Similar to previous results, the load–relative displacement graph increases linearly until a displacement of 1.5 mm and a load of 20 kN to 30 kN. At this point, the graph’s slope increases sharply, and the results continue linearly until a displacement of 5.5 mm and a load of 150 kN. Beyond this point, the slope of the graph slightly decreases until the average point of failure at 6.81 mm displacement and higher ultimate load than previous trials of 179.60 kN.

### 3.4. Confined PSB Connection (8.9 Mpa)

**Figure 10:**
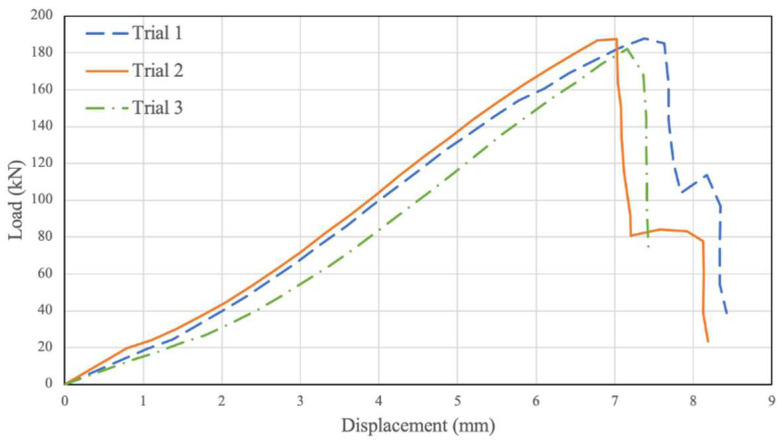
Confined 8.9 Mpa. Displacement vs. Load.

A further three trials were conducted when examining the behaviour of the PSB connections with the highest confinement pressure, 8.9 Mpa. The resulting data were plotted in Figure 10. As seen in other trials, the load–relative graph displacement initially increased linearly until a displacement of 1.5 mm and a load of 20 kN to 30 kN. After this point, the graph’s slope increased significantly, and the results continued linearly until a displacement of approximately 5.5 mm and loading between 130 kN and 160 kN. Beyond this point, the slope of the graph decreases until the average point of failure is 7.20 mm displacement with a final average connection load of 189.29 kN, which was the highest of all results recorded.

### 3.5. Summarised Experimental Results

**Figure 11:**
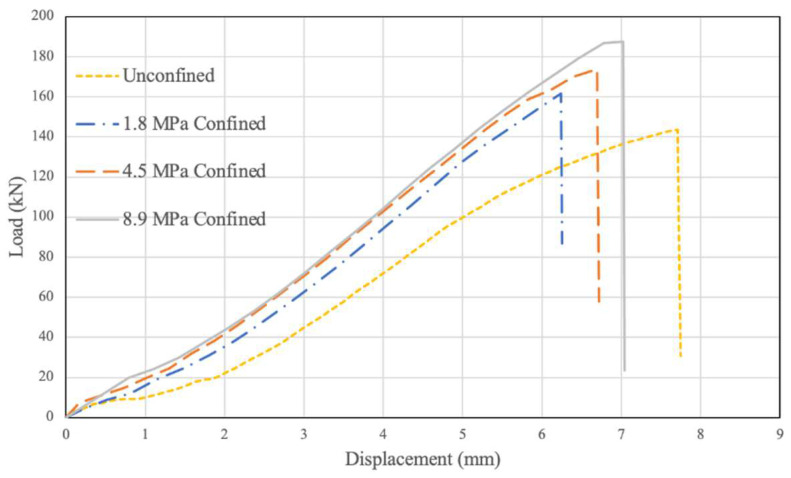
Combined Load vs. Displacement Results Under Various Levels of Confinement.

The experimental results for each confinement level can be summarised and compared by combining representative data trials into a multi-scatter plot. This is shown in Figure 11 below. The results highlight that the highest connection capacity was achieved under the highest level of confinement, 8.9 Mpa, followed by reduced capacities at 4.5 Mpa and 1.8 Mpa confinement. The lowest connection capacity occurred in the unconfined connections.

### 3.6. Failure Modes

**Figure 12:**
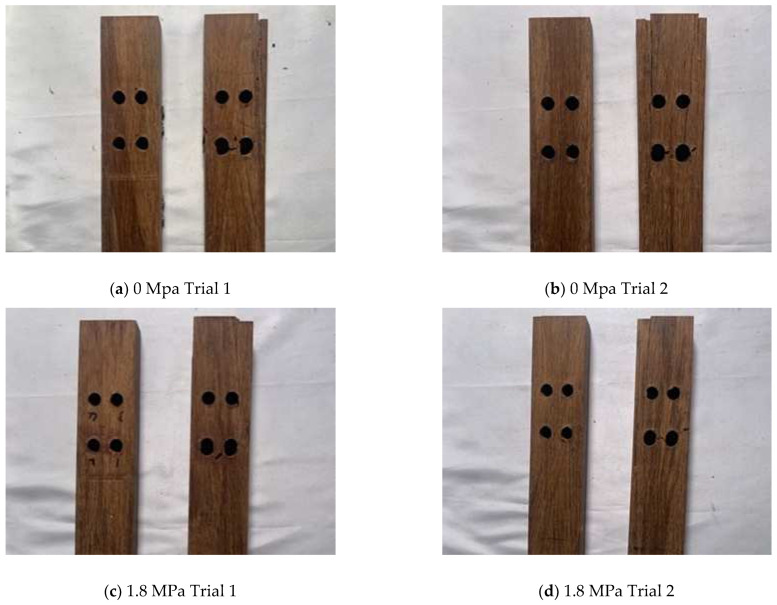

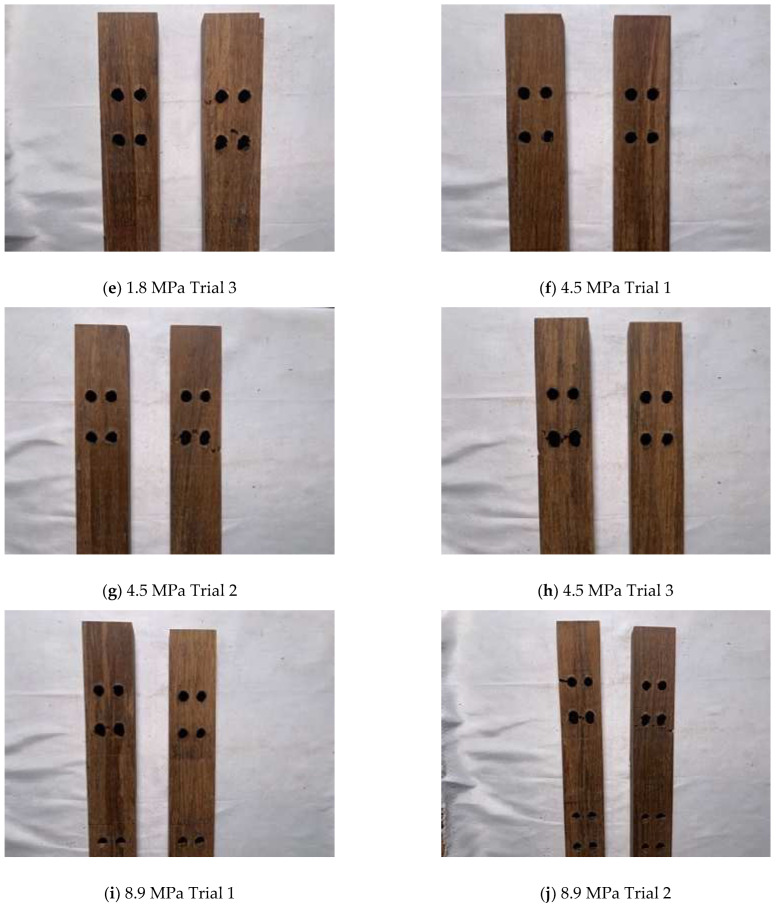

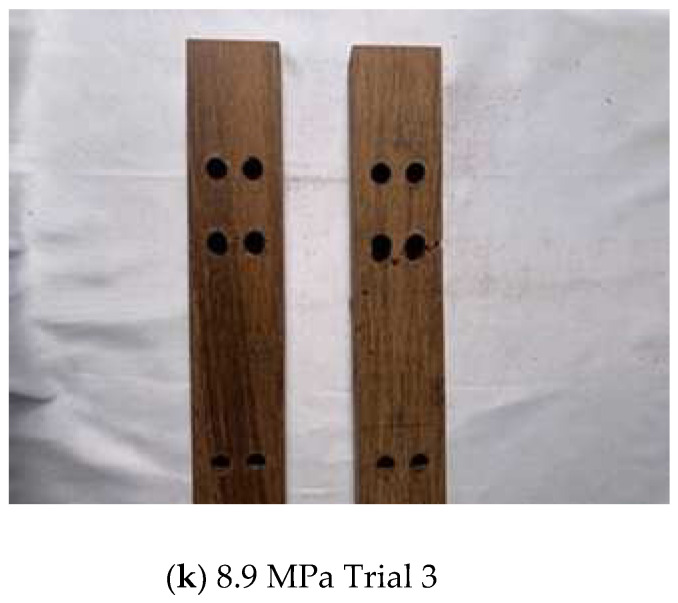
PSB Failure Modes.

A visual inspection of each failed PSB sample was conducted to identify the dominant failure mode and trends among the data. Each failed sample is shown in Figure 12, and the observed failure modes are tabulated in Table 2, along with each sample’s ultimate load and displacement.

Three different failure modes were observed across the trials: shear-out, bearing, and net-tensile failure. The predominant failure mode in the samples with no confinement was a shear-out failure, sometimes leading to net-tensile cracking following the initial shear crack. Similar to the first unconfined trial, all samples confined with 1.8 MPa experienced shear-out failure (with some consequent net-tensile cracking). However, these trials failed at greater ultimate loads than the unconfined connections. Conversely, when the confinement pressure increased to 4.5 MPa, the predominant failure mode shifted to pure net-tensile failure. One sample demonstrated bearing failure; however, this sample appears to be an outlier. As confinement pressure increased to 8.9 MPa, the predominant failure mode was consistently net-tensile failure, although with slightly higher ultimate loads than in previous trials.

## 4. ANSYS Numerical Simulation

**Figure 13:**
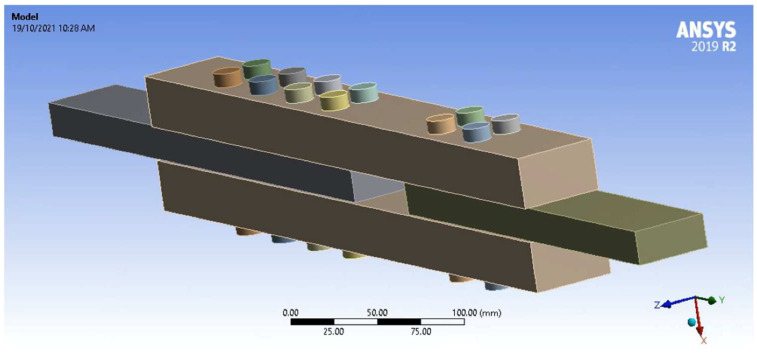
Unconfined Simulation Geometry.

An ANSYS numerical simulation model was built to re-produce the unconfined PSB connection experimental results and clarify experimental observations using finite element analysis software. The 2019R2 version of ANSYS was used, with the connection simulation geometry shown in Figure 13.

**Figure 14:**
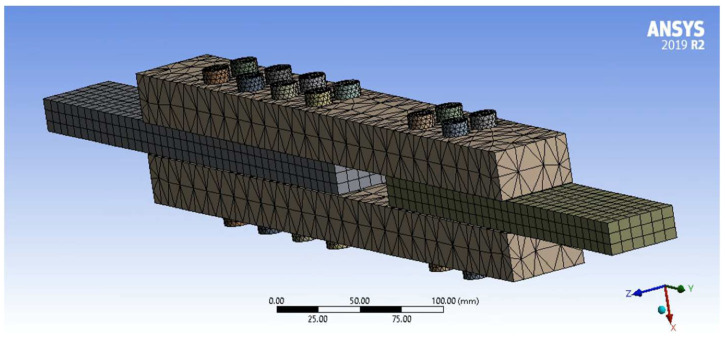
Simulation Mesh.

For the ANSYS simulation, PSB can be ideally treated as a transversely isotropic material since its bamboo strands are unidirectional and approximately uniformly distributed in the transverse direction [18]. The material properties used in the ANSYS simulation are outlined in Table 1. Various types of contacts were used in the ANSYS simulation depending on the physical context of geometry movement. The contacts between the inner surfaces of the bolt holes in the PSB and the steel bolt shanks were set to frictionless to allow separation in the normal direction and sliding in the tangential direction without frictional resistance [65]. For the same reason, the contacts between the inner surface of the steel plate bolt holes and the steel bolt shanks were also frictionless. The contacts between the PSB and steel surfaces were set as frictional contacts to allow separation in the normal direction and sliding in the tangential direction with some frictional resistance [65]. Finally, the contacts between the underside of the steel bolt nuts and the surface of the PSB were set to rough, which allows separation in the normal direction while disallowing sliding in the tangential direction (i.e., friction tends to infinity) [65]. Figure 14 shows the mesh used in the ANSYS simulation. The initial mesh was generated automatically for the PSB and steel plates with 8 mm element sizes and then refined further. A body sizing of 3 mm was applied to the steel bolts. Although the automatic mesh in the PSB is relatively coarse, a sensitivity analysis was completed to compare results from the coarse mesh to results from a more refined mesh. The difference between the results was insignificant, so the coarse mesh was adopted to reduce FEA computation time.

To best represent the boundary conditions in the experimental testing, fixed support was applied to the end surface of the far steel plate, and a displacement of 7 mm was applied to the end surface of the other steel plate in the second load step of the simulation. Bolt pretension was applied at a pretension load of 2000 N to each steel bolt in the connection to simulate the effect of 4 N.m of torque in the M10 bolts. The pretensioning setup consisted of linearly applying the 2000 N pretension load in the first load step, then locking the pretension load for the second load step while the displacement was applied.

**Figure 15:**
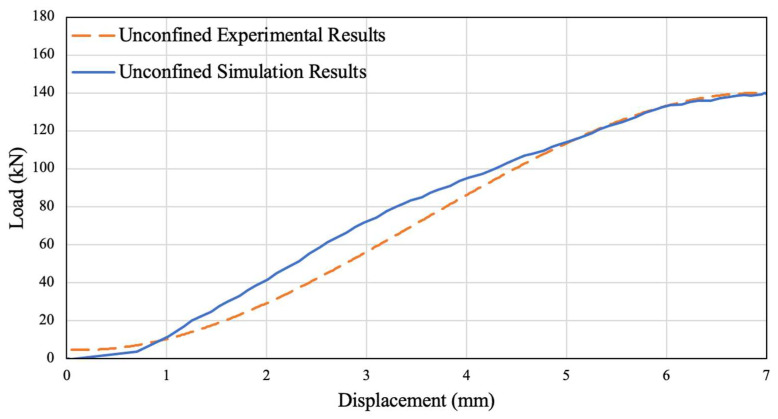
Unconfined Simulation and Experimental Results Comparison.

The simulation results are shown overlayed on the load vs. displacement graph of the unconfined experimental data in Figure 15. The comparison between simulated and experimental results shows that the simulation results are consistent with experimental data, thus verifying the numerical model.

## 5. Analysis and Discussion of Results

**Figure 16:**
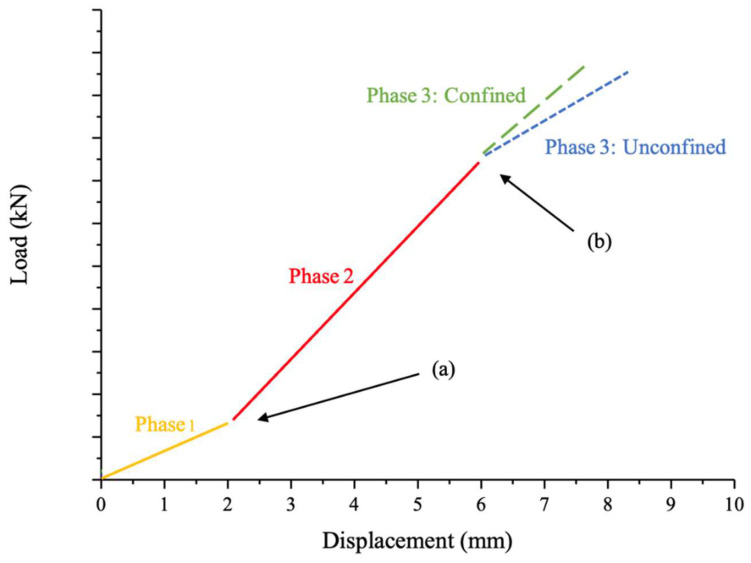
Generalised Phase Results. Phase 2 began at point (a) and demonstrated a significant increase in slope from Phase 1, suggesting an increase in connection stiffness under loading. This slope continued until the end of the phase at point (b).

Comparison of each graph of experimental results led to several implications. First, the beginning and end displacements of different sections of the graphs generally appeared to be consistent across the different levels of confinement. Secondly, there were similarities in the tensile loading values in some areas of the graph. Thirdly, the slopes of certain portions of each graph generally matched each other. These observations suggested that the behaviour of PSB bolted connections in the experiment could be broken into three general regions of the graph, denoted as phases, as annotated and shown in Figure 16. 

A summary of the approximate maximum connection loads at the end of each phase relative to the level of confinement is tabulated in Table 3, and the patterns and activities observed within each phase are discussed.

### 5.1. Phase 1—Independent of Confinement

**Figure 17:**
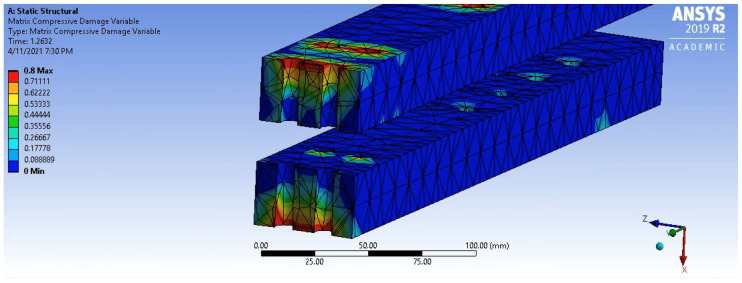
Matrix Compressive Damage at the End of Phase 1.

The Phase 1 portion of the graphs demonstrated linear behaviour in all experimental arrangements regardless of the presence of any confinement or confinement pressure. The maximum loads observed within this phase were consistently between 20 kN and 40 kN and occurred at point (a), with the confined experiments demonstrating slightly higher loads than unconfined. The slope of this phase showed minor variability between results, broadly suggesting independence of any confinement effect and consistency of material properties between experiments. At the end of Phase 1, damage analysis of the unconfined simulation model revealed the early onset of compressive matrix damage surrounding the bolt holes, as shown by the section plane in Figure 17.

This is due to the bolts compressing the material ahead of the bolt hole and the surrounding area, thus causing damage early in the loading process in this region. In contrast, matrix compressive damage in Phases 2 and 3 remained relatively constant until the point of failure. Although evidence suggests that compressive matrix damage is not the leading cause of failure in the connection, the Phase 1 compromise in matrix compressive strength occurring early in the loading process is likely to affect the ultimate strength of the connection. As such, it is hypothesised that mitigating the early onset of this damage may increase ultimate connection strength. This can be done by installing cover plates or washers underneath the bolts with appropriate pre-tensioning [58,59]; however, further experimentation is required to verify this hypothesis.

### 5.2. Phase 2—Independent of Confinement

In Figure 16 phase 2 began at point (a) and demonstrated a significant increase in slope from Phase 1, suggesting an increase in connection stiffness under loading. This slope continued until the end of the phase at point (b). The fact that the increased slope at the beginning of this phase occurred at the same approximate point in all experimental tests demonstrates that, similar to Phase 1, this phase was also independent of confinement and confinement pressure.

### 5.3. Phase 3—Influenced by Confinement and Damage

**Figure 18:**
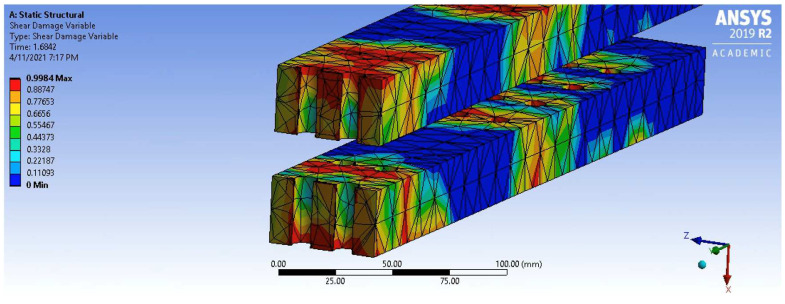
Shear Damage Status at the Beginning of Phase 3.

Contrary to Phases 1 and 2, Phase 3 was influenced by connection confinement and its impact on damage evolution. It was seen that the unconfined PSB connection behaved differently than all confined connections in this phase. Phase 3 began at point (b); however, the slopes of the unconfined and confined connection graphs diverged during the phase. In the unconfined connection, Phase 3 decreased to a similar but slightly larger, slope than Phase 1. This reflects a decrease in overall connection stiffness, which is likely due to the evolution of shear damage, as suggested by the simulation results. Analysis and comparison of the damage parameters in the unconfined connection simulation model revealed that the parameter most significantly leading to the reduction in stiffness before failure in Phase 3 was shear damage. The shear damage status at the beginning of Phase 3 is shown in Figure 18, where a section plane was taken through the bolt hole to reveal the extent of internal shear damage in the PSB at the points of highest stress concentration.

**Figure 19:**
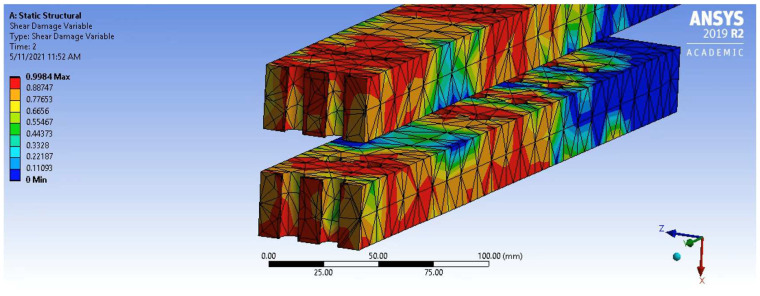
Shear Damage Status at Failure.

It can be seen that shear damage is significant around the surface of the bolt hole and the surrounding regions but reduces variably away from this point and is still relatively low in other areas. Although shear damage evolves throughout Phase 1 and 2, the impact of this damage evolution was not truly seen until the beginning of Phase 3, where connection stiffness was compromised, and the slope of the unconfined force–displacement graph decreased until ultimate failure. The final shear damage status of the unconfined connection at failure is shown in Figure 19. Since the start of Phase 3, shear damage in the PSB evolved significantly, and most of the cross-section was completely compromised. As a result, the stiffness and strength of the connection reduced considerably to the point where the failure occurred due to a combination of excess shear stress.

**Figure 20:**
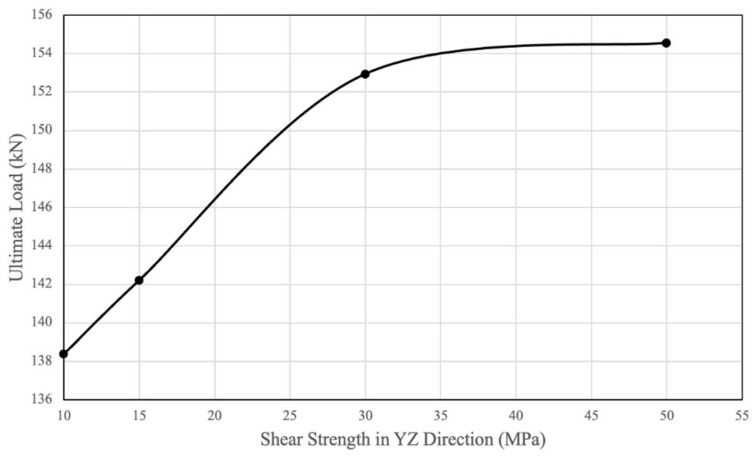
Impact of YZ Shear Strength on Ultimate Load.

To examine the effect of shear strength on the strength of PSB bolted connections, a sensitivity analysis of PSB shear parameters was conducted. The YZ shear stress limit parameter (YZ orientation can be seen in Figure 5) was adjusted and resulted in the observation that the ultimate tensile load capacity of the connection increased as the YZ shear strength parameter increased. As Figure 20 shows, there is an approximately linear relationship between YZ shear strength and ultimate tensile capacity with an upper bound of approximately 155 kN when shear capacity increases beyond 35 MPa. The original values of shear stress limits are presented in Table 1.

**Figure 21:**
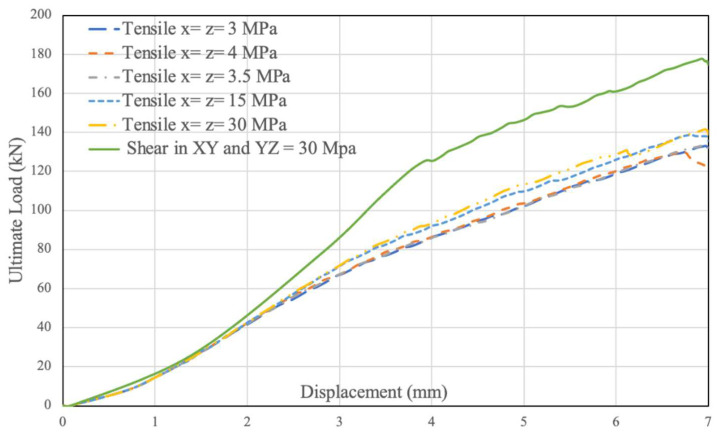
Impact of Tensile and Shear Strength on Ultimate Load.

Further sensitivity analysis using the unconfined connection model increased both shear stress limits YZ and XZ to 30 MPa, which resulted in a large increased ultimate capacity of approximately 180 KN as shown in Figure 21. In contrast to shear capacity, the tensile capacity perpendicular to the fibres, i.e., in the X and Z directions (see Figure 5 for axis legend), was found to have an insignificant effect on the ultimate capacity of the connection. Figure 21 also shows force relative displacement simulation results for a range of tensile capacities, from 3 to 30 MPa. These results highlight the minimal impact of tensile strength and the major impact of XY and YZ shear strength on ultimate load.

Results of the sensitivity analyses suggest that increasing tensile capacity perpendicular to the PSB fibres in the testing of the unconfined connections would not improve the ultimate capacity of the connection. This also suggests that the failure of the tested connections is unlikely to be due to cleavage mode since cleavage failure is sensitive to tensile stress limits perpendicular to the fibres (i.e., in the X and Z direction) while the results show an insignificant change in force–displacement diagrams when changing the tensile stress limits in the X and Z directions. However, changes in the shear stress limits resulted in significant changes in the force–displacement diagrams, which suggests that the tested unconfined connection is sensitive to changes in shear capacity and, as a result, is likely to fail in the shear-out mechanism. This observation is aligned with experimental failure mechanisms, which showed the unconfined connections tested failed in shear-out mode. The observations regarding the impact of shear strength on the unconfined connection ultimate capacity highlight the possibility of increasing the viability of these PSB connection setups by increasing the shear strength of the PSB material.

On the other hand, the confined connections experienced a different pattern in Phase 3 compared to the unconfined connections. In all levels of confinement pressure, the slope of the graph continued relatively unchanged between Phases 2 and 3 until the point of failure, indicating a minimal reduction in stiffness and load-carrying capacity in this phase despite the increasing load. This is hypothesised to be due to the tendency for confinement to delay the onset of damage by reducing the net tensile and shear stresses in the PSB. As a result, the material properties are largely maintained until excessive longitudinal tensile stresses cause fibre damage and sudden rupture when a net-tensile failure occurs. 

Although the specific force values differed between different levels of confinement, this general pattern was experienced regardless of confinement pressure, suggesting that generally, confinement tends to delay the onset of material damage, maintain material stiffness properties, and increase ultimate tensile capacity until sudden failure. However, further research into damage progression throughout the loading process is necessary to verify this phenomenon in confined connections. Furthermore, a trend was observed in Phase 3, where higher connection ultimate tensile capacity was experienced under higher confinement pressure. The experimental results revealed that 1.8 MPa of confinement pressure resulted in an average ultimate load of 162 kN. In comparison, 4.5 MPa of pressure increased the average ultimate tensile load to 179 kN, and 8.9 MPa of pressure increased the average ultimate tensile load to 189 kN.

The percentage increase in maximum load at different levels of confinement pressure relative to the unconfined connection ranged from a minimum of 7.5% capacity increase for the 1.8 MPa confined connections, 19.2% capacity increase for the 4.5 MPa confined connections, and as high as 25.7% capacity increase for the 8.9 MPa confined connections. This suggests a relationship between confinement pressure and connection tensile capacity, whereby an increase in confinement pressure increases connection tensile capacity. These observations are supported by previous case studies of confinement methods [62,64].

### 5.4. Stiffness Increase

A summary of the effect on connection stiffness related to damage during each graphical phase is outlined in Table 4 below.

The phenomenon of an increase in stiffness in Phase 2 is a pivotal contributor to the ultimate load-carrying capacity of the connections. If the slope in Phase 1 in the experimental load vs. displacement graphs is extrapolated, it becomes clear that the connection would not achieve even half of the ultimate capacity experienced without the increase in stiffness in Phase 2. However, it was noted that this Phase 2 stiffness increase was present in all experimental connection setups, indicating the phenomenon was consistent regardless of the presence of a confining force and is therefore not an effect of confinement. Moreover, the simulation highlights that damage evolves over Phase 2; thus, the increase in stiffness occurred despite damage developing.

### 5.5. Failure Mode

The results also demonstrated a trend in the effect of confinement on the predominant mode of connection failure. In Table 2, it was highlighted that the dominant mode of failure for samples with no confinement was shear-out with some subsequent net-tensile cracking. The confined connections with 1.8 MPa of pressure also failed in shear-out followed by net-tensile cracking; however, the ultimate connection loads at the point of failure for the 1.8 MPa connections were higher than the unconfined connections. Contrastingly, the predominant failure mode for samples confined with 4.5 MPa and 8.9 MPa of pressure was net-tensile failure with one outlier of bearing failure. These results are consistent with previous hypotheses. Since the shear strength parallel to the fibres is three times less than perpendicular to the fibres [18], shear-out failure occurs when there is no additional normal stress perpendicular to the fibres to increase shear capacity. This failure mode predominantly occurred in the unconfined and 1.8 MPa confined connection trials.

On the other hand, net-tension failure results from excessive tensile stresses along the axis of fibre orientation, and this was the predominant mode of failure of the 4.5 MPa and 8.9 MPa confined connections. Since the tensile strength of PSB parallel to the fibres is three times higher than perpendicular to the fibres [18], the ability of the connection load to increase to the point of reaching the maximum axial tensile strength of the fibres signifies that parallel shear stresses and perpendicular tensile stresses were relatively low and did not play a significant part in the failure of these samples with higher confinement pressures. This further supports the hypothesis that confinement induces lateral pressure, which opposes tensile forces and improves the shear capacity of the PSB connections since there were no significant resultant shear stresses along the axis of fibre orientation that might cause shear-out failure in these samples. 

It is also interesting to note the levels of confinement at which the shift occurred from the predominant shear-out mode of failure to the net-tensile mode of failure. Since higher levels of confinement resulted in more consistent net-tension failure and less shear-out failure, the trend indicates that higher levels of confinement tend to impose additional normal pressure perpendicular to the fibre orientation more effectively, thereby more effectively preventing shear-out. The exceptions to this trend are the samples with the lowest level of confinement at 1.8 MPa, which still experienced shear-out failure despite being confined. This is likely due to the relatively low confinement pressure induced onto the PSB, which was insufficient to counteract the shear stresses generated during loading. The analysis of the failure modes of PSB bolted connections supports the hypothesis that confinement is an effective method of opposing parallel shear stresses and thus generating larger shear capacity in PSB. By opposing these stresses, the results suggest that confinement delays the shear-out failure with low levels of confinement and prevents the shear-out failure with higher levels of confinement.

## 6. Conclusions

This study investigated the effect of connection confinement on the strength and failure mode of bolted connections made from a bamboo composite material known as PSB. Four levels of connection confinement pressure were tested under tensile load until failure: 0 MPa (i.e., unconfined), 1.8 MPa, 4.5 MPa, and 8.9 MPa. The load–relative displacement graphs of each experimental test were generalised into three distinct graph phases, as shown in Figure 16. Graphical comparison of these phases, as well as observations regarding failure mode, resulted in the following highlighted findings:

- Unconfined connections tend to fail in shear-out failure mode due to excessive shear stresses parallel to the PSB fibre orientation axis, which leads to matrix cracking.
- Relatively low levels of connection confinement tend to delay the onset of shear-out failure (but not prevent it) and allow the connection to achieve higher ultimate capacity.
- Relatively high levels of connection confinement sufficiently prevent shear-out failure by providing lateral compressive stress that opposes shear stresses developed parallel to the axis of fibre orientation.
- Highly confined connections tend to fail in net-tensile failure mode due to fibre rupture as the prevention of shear-out failure allows the connection fibres to reach their ultimate tensile capacity.
- A sensitivity analysis using the unconfined connection simulation highlighted that increases in the shear strength of the PSB material resulted in increases in the ultimate tensile load capacity of connections.
- The ultimate capacity of the tested PSB confined connections increased by up to 26% as the confinement pressure increased. This led to the conclusion that as the level of confinement increases, the ultimate connection tensile capacity also increases.
- Finally, it was observed that connection confinement maintains connection stiffness better than unconfined connections during phases with high loads. This is likely due to the ability of confinement to delay the onset of damage within the PSB.

## Figures and Tables

**Table 1:** Materials used for the preparation of the samples.

Mechanical Property	Mean Value
Elastic Modulus Y	10.30 GPa
Elastic Modulus X, Z	2.22 GPa
Shear Modulus XY	1.45 GPa
Shear Modulus YZ	1.36 GPa
Shear Modulus XZ	0.746 GPa
Tensile Strength Y	118.40 MPa
Tensile Strength X, Z	4.43 MPa
Compressive Strength Y	65.53 MPa
Compressive Strength X, Z	23.14 MPa
Shear Strength XY	8.21 MPa
Shear Strength YZ	8.21 MPa
Shear Strength XZ	3.64 MPa
Poisson’s Ratio	0.3

**Table 2:** Summary of Test Results.

Predominant Failure Mode	Average Ultimate Load (kN)	Average Displacement at Ultimate Load (mm)
**No Confinement**		
Shear-Out	150.61	7.2
**1.8 MPa Confinement Pressure**		
Shear-Out	161.92	7.17
**4.5 MPa Confinement Pressure**		
Net-Tensile	179.6	6.81
**8.9 MPa Confinement Pressure**		
Net-Tensile	189.29	7.2

**Table 3:** Summary of Final Tensile Load in Each Phase.

	Final Load (kN)			
Confinement Level	0 MPa	1.8 MPa	4.5 MPa	8.9 MPa
Phase 1	20	30	35	35
Phase 2	150	150	150	160
Phase 3	155	170	180	190

**Table 4:** Summary of Phase Stiffness Changes.

	Unconfined	Confined
Phase 1	Initial Stiffness—No damage effect	Initial Stiffness—No damage effect
Phase 2	Stiffness Increase—Damage evolving but no effect seen	Stiffness Increase—Damage evolving but no effect seen
Phase 3	Stiffness Decrease—Effect of damage seen	Stiffness Mostly Maintained—Minor effect of damage with most damage delayed

## Data Availability

Not applicable.
